# Supplementary material for: Feline Oral Squamous Cell Carcinoma: A Critical Review of Etiologic Factors
Source: Vet Sci. 2022 Oct 11;9(10):558. doi: 10.3390/vetsci9100558 (PMC9609408; doi:10.3390/vetsci9100558)
Supplement: Supplementary file 1 [file vetsci-09-00558-s001.zip › vetsci-1869759-supplementary.pdf]

veterinary sciences

MDPI

Supplementary Material

Feline Oral Squamous Cell Carcinoma: A Critical Review of Etiologic Factors

Inês Sequeira, Maria dos Anjos Pires, José Leitão , Joaquim Henriques, Carlos Viegas and João Requicha

Table S1. Grading system used to score the quality of experimental design for manuscripts included in this systematic review, modified from a grading system published by the Oxford Centre for Evidence-based Medicine.

Experimental design grade (EDG)	Study types
I	Randomized, controlled, double-blinded prospective clinical trials
II	Prospective clinical trials
III	Retrospective case series, (n > 10)
IV	Retrospective case series (n < 10)
V	Single patient case report; expert opinion

.

Table S2. Data collection related to viruses.

Reference	Geographic location	Level of evidence	Cats with FOSCC (n)	Breed (n)	Gender (n)	Animals' lifespan (n)	Virus (% positive)	Diagnostic test
Chu *et al*., 2019 [34]	USA	III	20	ND	M (10)<br>F (10)	AdMa (8)<br>G (12)	FcaPV-3 (5%)<br>FFV (35%)<br>FTTV (10%)<br>EBV (5%)<br>FHV-1 (10%)<br>FeLV (0%)<br>FIV (0%)	ViroCap, PCR (only on EBV and FcaPV – 3 positive)
Gendler *et al*., 2010[47]	USA	III	18	ES (14)<br>H (2)<br>MC (2)	M (7)<br>F (11)	13.2 ± 3.5	FIV (0%)<br>FeLV (0%)	Only referred sanitary status
Yamashita *et al*., 2018 [32]	Japan	IV	8	ES (8)	M (4)<br>F (4)	Sen (5)<br>G (3)	FcaPV-2 (0%)<br>FcaPV-3 (0%)<br>FcaPV-4 (0%)	PCR; IHC p16
Altamura *et al*., 2020 [35]	Italy	III	32	ES (18)<br>Per (1)<br>SI (13)	M (6)<br>F (15)<br>ND (11)	Ad (1)<br>AdMa (5)<br>Sen (6)<br>G (1)<br>SI (19)	FcaPV-2 (31,3%)	PCR; IHC (only on PV-positive)
O'Neill *et al*., 2011 [24]	USA	IV	2	ES (1)<br>H (1)	M (1)<br>F (1)	AdMa 1)<br>G (1)	HVP-38 (25%)<br>PV (25%)	PCR
Munday *et al*., 2009 [29]	New Zealand	III	20	EC (1)<br>SI (19)	ND	AdMa (1)<br>ND (19)	PV (5%)	PCR

Munday *et al.*, 2011 [30]	New Zealand	III	30	ND	ND	12.8	PV (0%)	PCR; IHC p16
Altamura *et al.*, 2018 [27]	Italy	IV	1	EC (1)	ND	Sen (1)	FcaPV-2 (100%)	PCR; IHC p53 and E6AP
Munday *et al.*, 2015 [17]	New Zealand	III	36	ND	ND	AdMa (1)<br>Ad (1)<br>SI (34)	FcaPV-1 (2.8%)<br>FcaPV-4 (0%)	PCR; IHC p16 (only on FcaPV-1 positive)
Munday *et al.*, 2019[23]	New Zealand	III	52	ND	M (32)<br>F (20)	13.5	PV (0%)	IHC p16 and p53 and PCR (only on p16-positive)
Hoggard *et al.*, 2018 [33]	USA	IV	1	EC (1)	ND	Sen (1)	FcaPV-2 (0%)	PCR; ISH
Anis *et al.*, 2010[36]	USA	IV	1	ND	ND	ND	HVP-38 (100%)	PCR
Hutson *et al.*, 1991[72]	USA	IV	18[a]	ND	ND	ND	FIV (22.2%)	PCR
Supsavhad *et al.*, 2016[31]	USA	III	43	ES (37)<br>H (3)<br>S (1)<br>Per (1)<br>A (1)	M (16)<br>F (27)	J (1)<br>Ad (3)<br>AdMa (5)<br>Sen (20)<br>G (13)	PV (0%)	IHC; PCR (only on p16-positive)
Olmsted *et al.*, 2016 [55]	USA	III	35	ES (31)<br>Ab (1)<br>B (1)<br>MC (1)	M (16)<br>F (19)	14	FIV (2.9%)	Only referred sanitary status

Altamura *et al*., 2016 [26]	Italy	IV	1	ES (1)	ND	Sen (1)	FcaPV-2 (100%)	PCR; IHC (p53 and pRb)
Altamura et al., 2022 [37]	Italy	III	113	ES (113)	ND	ND	FcaPV-1 (6.2%)<br>FcaPV-2(7.5%)<br>FcaPV-3(5.3%)<br>FcaPV-4(0.9%)<br>FcaPV-5(1.8%)	PCR
Zaccone *et al*., 2021 [73]	Italy	III	100	Ch (5)<br>S (2)<br>P (1)<br>MC (1)<br>K (1)<br>ES (90)	M (46)<br>F (54)	ND	FeLV (0%)<br>FIV (8%)	Only referred sanitary status

M – Male; F – Female; PV – Papilomavirus; IHC – Immunohistochemistry; PCR – Polymerase chain reaction; RT-PCR – Reverse transcription PCR; ISH – *in situ* hybridization; FcaPV – *Felis catus* papillomavirus; FFV – *Feline foamy virus*; FTTV – *Feline torque teno virus*; EBV – *Epsteinn-Barr virus*; FHV – Feline herpesvirus; FIV – Feline immunodeficiency virus; FeLV – *Feline Leukemia virus*; HPV – Human Papillomavirus; H – Himalayan; ES – European Shorthair; S – Siamese; A – Angorá; Pe – Persian; Ab – Abyssinian; B – Bengal; J – Júnior; Ad – Adult; AdMa – Adult Mature; Sen – Senior; G – Geriatric; ND – No data; USA – United States of America. [a] – 18 cats with FOSCC without specific location.

Table S3. Data collection related to environmental tobacco smoke.

Reference	Geographic location	Level of evidence	Cats with FOSCC (n)	Breed (n)	Gender (n)	Animals' lifespan (n)	Exposure to EST (% of animals; RR; *p*)
Bertone *et al.*, 2003 [14]	USA	III	36	ES (34)<br>Purebred (2)	M (16)<br>F (20)	AdMa (4)<br>Sen (21)<br>G (11)	E (36.1%; 2.3; 0.11)<br>NE (61.1%; 1.0; -)
Snyder *et al.*, 2004 [12]	USA	III	23	ND	M (16)<br>F (17)	ND	E (47.8%; 4.5; 0.19)<br>NE (52.2%; 1.0; -)
Renzi *et al.*, 2019 [13]	Italy	III	24[b] (26)	ES (22)<br>Ch (2)<br>S (1)<br>Per (1)	M (12)<br>F (14)	14.5	E (25.0%; -; -)<br>NE (75.0%; -; -)
Zaccone *et al.*, 2021[73]	Italy	III	100	Ch (5)<br>S (2)<br>P (1)<br>MC (1)<br>K (1)<br>ES (90)	M (46)<br>F (54)	ND	E (31%; -; -)<br>NE (69%; -; -)

ES – European Shorthair; Ch – Chartreux; S – Siamese; Persa – Persian; M – Male; F – Female; E – Exposed; NE – Not exposed; P – Positive; N – Negative; Ad – Adult; AdMa – Adult Mature; Sen – Senior; G – Geriatric; ND - No data; RR – Relative risk; *p* – significance level; USA – United States of America. [b] Information about smoke exposure is only available in 24 out of 26 cats.

Table S4. Data collection related to oral comorbidities and other risk factors.

Reference (author, year of publication)	Geographic location	Level of evidence	Cats with FOSCC (n)	Breed (n)	Gender (n)	Animals' lifespan (n)	Flea control product (% animals; RR, *p*)	Diet (% animals; RR; *p*)	Oral comorbidities (% animals; RR; *p*)	Other risk factors (% animals; RR; *p*)
Bertone *et al.*, 2003 [14]	USA	III	36	ES (34) Pure (2)	M (16) F (20)	AdMa (4) Sen (21) G (11)	Never (38.9% 1.0;-) Collar (36.1%; 5.3; 0.002) Shampoo (5.6%; 0.1; 0.011) Powder (13.9%; 1.7; 0.42) Spray (13.9%; 1.2; 0.81)	Dry (27.7%;1.0;-) Canned (55.5%; 3.6; 0.014) Dry and canned (16.7%; 2.2; 0.27) Canned tuna (41.7%; 4.7; 0.004) Table scraps (44.4%; 1.2; 0.87) Liver (2.8%; 0.3; 0.30)	Dental pathology (33.3%; 1.8; 0.18)	City (13.9%; 0.4; 0.067) Small town (55.5%; 1.0; -) Rural (27.7%; 0.7; 0.52) WS city (33.3%; 1.0: -) Other WS (63.9%; 0.6; 0.23) HS gas (2.8%; 0.4; 0.12) HS wood (30.6%; 0.8; 0.65) HS oil (19.4%; 0.8; 0.61) HS coal (61.1%; 0.9; 0.90) Grooming (7%; 1.3; 0.58) Bathing (2%; 0.5; 0.16)
Snyder *et al.*, 2004 [12]	USA	III	23	-	M (16) F (17)	ND	Never (34.8%; 1.0)	Dry (21.7%; 1.0)	-	-

							Collar (43.5%; 0.7; 0.21) Shampoo (0) Other (21.7%; -; -)	Canned (43.49%; 0.7; 0.99) Canned tuna (52.2%; 1.1; 0.99)		
Pavlin *et al.*, 2018 [56]	Slovenia	V	1	ES (1)	M (0) F (1)	Sen (1)	-	-	Periodontal disease; Tooth resorption; Dental loss (100%)	Dental plaque
Olmsted *et al.*, 2016 [55]	USA	III	35	ES (31) Ab (1) B (1) MC (1) BN (1)	M (16) F (19)	14	-	-	Feline chronic gingivostomatitis (2.8%)	-
Hayes *et al.*, 2007 [6]	United Kingdom	III	54	ES (50) MC (1) P (1) Bu (1)	M (29) F (25)	13.3	-	-	Dental pathology (31%)	-
Boston *et al.*, 2018 [74]	USA	II	7	ES (6) BN (1)	M (4) F (3)	AdMa (1) Sen (3) G (3)	-	-	Dental loss (14.3%)	-
Quigley *et al.*, 1972[68]	United Kingdom	IV	4	-	M (2) F (2)	AdMa (1) Sen (3)	-	-	Osteosarcoma (50%)	Dental residues

Moisan *et al.*, 1998 [54]	USA	V	1	ES (1)	M (1)	AdMa (1)	-	-	*Trichinella* spp. (100%)	-
Zaccone *et al.*, 2021 [73]	Italy	III	100	Purebreed (10%)<br>ES (90%)	M (46)<br>F (54)	ND	Collar (3%;-;0.01)<br>Oral products (4%;-;-)<br>Spot - on drops (59%;-;-)	Dry (32%)<br>Canned tuna (63%)<br>Table scraps (20%)<br>Food with high chemical additives(80;-;0.01)	Oral inflammation (34%)	Trivalent vaccine occasional (52%)<br>Trivalent vaccine regular (36%)<br>Cohabitation with other cats (63%)<br>Rural living envionment(37%;-;0.04)

ES – European Shorthair; S – Siamese; P – Persian; M – Male; F – Female; MC – Main Coon; BQ – Norwegian Forest Cat; B – Bengal; Bu – Bumese; Ab – Abyssinian; AdMa – Adult Mature; Sen – Senior; G – Geriatric; HS – Heat Source; WS – Water Source; RR – Relative risk; *p* – significance level; USA – United States of America. [c] – Possibility of choosing more than one option.

Table S5. Results of quality assessment using Downs and Black score.

Reference	Score	Reference	Score
Chu *et al*., 2019[34]	7	Altamura *et al*., 2018 [27]	4
Gendler *et al*.,2010 [47]	6	Munday *et al*., 2015 [17]	3
Yamashita *et al*., 2018 [32]	6	Munday *et al*., 2019 [23]	5
Altamura *et al*., 2020 [35]	6	Hoggard *et al*., 2018 [33]	5
O'Neill *et al*., 2011 [24]	5	Anis *et al*., 2010 [36]	4
Munday *et al*., 2009 [29]	4	Hutson *et al*., 1991 [72]	4
Munday *et al*., 2011 [30]	5	Supsavhad *et al*., 2016 [31]	3
Olmsted *et al*., 2016 [55]	8	Renzi *et al*., 2019 [13]	6
Altamura *et al*., 2016 [26]	5	Pavlin *et al*., 2018 [56]	5
Bertone *et al*., 2003 [14]	6	Hayes *et al*., 2007 [6]	6
Snyder *et al*., 2004 [12]	5	Boston *et al*., 2018 [74]	6
Quigley *et al*., 1972 [68]	4	Altamura *et al*., 2020[35]	7
Moisan *et al*., 1998 [54]	4	Zaccone *et al*., 2021 [73]	6
